# Real-time assessment of cigarette smoke particle deposition *in vitro*

**DOI:** 10.1186/1752-153X-6-98

**Published:** 2012-09-10

**Authors:** Jason Adamson, Sophie Hughes, David Azzopardi, John McAughey, Marianna D Gaça

**Affiliations:** 1 British American Tobacco, Group R&D, Regents Park Road, Southampton, SO, 15 8TL, UK

**Keywords:** QCM, Cigarette smoke, *In vitro* exposure chamber, Dosimetry

## Abstract

**Background:**

Recently there has been a rapid increase in approaches to assess the effects of cigarette smoke *in vitro*. Despite a range of gravimetric and chemical methods, there is a requirement to identify simpler and more reliable methods to quantify *in vitro* whole smoke dose, to support extrapolation and comparisons to human/*in vivo* dose. We have previously characterised an *in vitro* exposure system using a Borgwaldt RM20S smoking machine and a chamber exposing cellular cultures to whole smoke at the air-liquid interface. In this study we demonstrate the utility of a quartz crystal microbalance (QCM), using this exposure system, to assess real-time cigarette smoke particulate deposition during a 30 minute smoke exposure. Smoke was generated at various dilutions (1:5–1:400, smoke:air) using two cigarette products, 3R4F Kentucky reference and 1 mg commercially available cigarettes. The QCM, integrated into the chamber, assessed particulate deposition and data generated were compared to traditional chemical spectrofluorometric analysis.

**Results:**

The QCM chamber was able to detect mass differences between the different products within the nanogram range. 3R4F reference cigarette smoke deposition ranged from 25.75 ±2.30 μg/cm^2^ (1:5) to 0.22 ±0.03 μg/cm^2^ (1:400). 1 mg cigarette smoke deposition was less and ranged from 1.42 ±0.26 μg/cm^2^ (1:5), to 0.13 ±0.02 μg/cm^2^ (1:100). Spectrofluorometric analysis demonstrated statistically significant correlation of particulate deposition with the QCM (p < 0.05), and regression R^2^ value were 97.4 %. The fitted equation for the linear model which describes the relationship is: QCM = −0.6796 + 0.9744 chemical spectrofluorescence.

****Conclusions**:**

We suggest the QCM is a reliable, effective and simple tool that can be used to quantify smoke particulate deposition in real-time, *in vitro* and can be used to quantify other aerosols delivered to our chamber for assessment.

## Background

Cigarette smoke is a complex and dynamic aerosol consisting of at least 5,600 chemicals and toxicants found across two phases, the particulate (tar) and vapour phase [1]. Recently, there has been a rapid increase in the development of systems for *in vitro* biological and toxicological assessment of whole smoke [2-11]. However, despite these advancements there have not been consistent approaches in reporting accurately the dose of whole smoke delivered to *in vitro* cultures.

Understanding dosimetry is essential when attempting to mimic or extrapolate human smoking behavior and *in vivo* doses to *in vitro* models. Whole smoke dose is dependent on the machine used to generate, dilute and deliver smoke and is variously described as a percentage of smoke, a fraction of smoke, ratios of smoke to air, puff number, total exposure of micrograms per insert, or as a flow rate of mixing air and vacuum applied to a smoke dilutor [2,3,5,6,9-11]. This is a relatively new and challenging field but is an increasingly important point of discussion within the industry. On a broader note, the need to quantify absolute chemical or particle deposition in *in vitro* model systems is of increasing importance to scientists and regulators for consistent interpretation of disease model end-points versus a defined biologically effective dose [12,13].

There are a number of reported studies quantifying components of either the particulate or vapour phase as a means of assessing dose. Solanesol is the most common constituent measured in the particulate phase [14], and carbon monoxide in the gas phase [6]. Most dosimetry measurements of cigarette smoke are of the particulate phase due to the challenges of measuring individual components in the vapour phase, especially at higher smoke dilutions. However, many of the methodologies involved are complex, often off-line and involve many steps where errors or loss of precision could be introduced, and there is no general consensus on the most appropriate approach. There is therefore a requirement for a simple, more reliable and a standard method to be used for whole smoke *in vitro* dose assessments.

The quartz crystal microbalance (QCM) is a sensitive gravimetric balance capable of measuring and detecting changes in mass, within the nanogram range, of thin adherent films [15-17], and has been used as such since the 1950’s following pioneering scientific work by Sauerbrey [18]. It makes use of the piezoelectric effect associated with all quartz crystals. Mechanical and electrical stress applied to the crystal, when incorporated into an electrical circuit, produces an electric potential, and when applied to the crystal produces mechanical deformation on the crystal [16,19]. These properties, when employed, generate waves whose frequencies are influenced by changes in mass at the crystal surface [20].

The QCM consists of a thin quartz disc held between two electrodes, often made of gold, combined with software technology capable of monitoring and recording changes in frequency. The rate of oscillation of the quartz crystal is directly related to its thickness (when other variables such as temperature and humidity remain constant), therefore crystals of the same specific thickness will oscillate at the same resonant frequency [19,21]. As mass is added onto an oscillating quartz crystal, its effective thickness is increased. This change in thickness correlates directly to a change in oscillation frequency: the greater the deposition of a given substance onto the crystal surface, the lower the frequency of oscillation [21]. Sauerbrey’s equation [18] can be employed to convert the frequency shift into the mass per unit area of thin film deposition [16,19]. Under ideal conditions, it is assumed that the deposited mass forms a monolayer, hence changing the effective thickness of the crystal as described with the deposited mass fully coupled to the crystal. In practice, the smoke particles are approximately 300 nm count median diameter (cmd), and while not initially forming a monolayer, they are sufficiently small that they would not be expected to oscillate independently of the crystal.

QCMs have been used for a wide variety of applications, one of the most common being the monitoring of water pollution [17]. Many advances have also been made in biological disciplines where QCMs have been used to detect entities as small as virus nanoparticles [22] and peptide membrane binding dynamics [23]. QCMs have also been utilised to quantify different types of smoke by mass, such as outdoor tobacco smoke [24] and blood and bone associated aerosols/cautery smoke from orthopaedic surgery [25], as well as ultrafine particles *in vitro*[15]; however, to our knowledge a QCM has not yet been reported to quantify cigarette smoke dosimetry *in vitro* within an exposure chamber.

**Figure 1:**
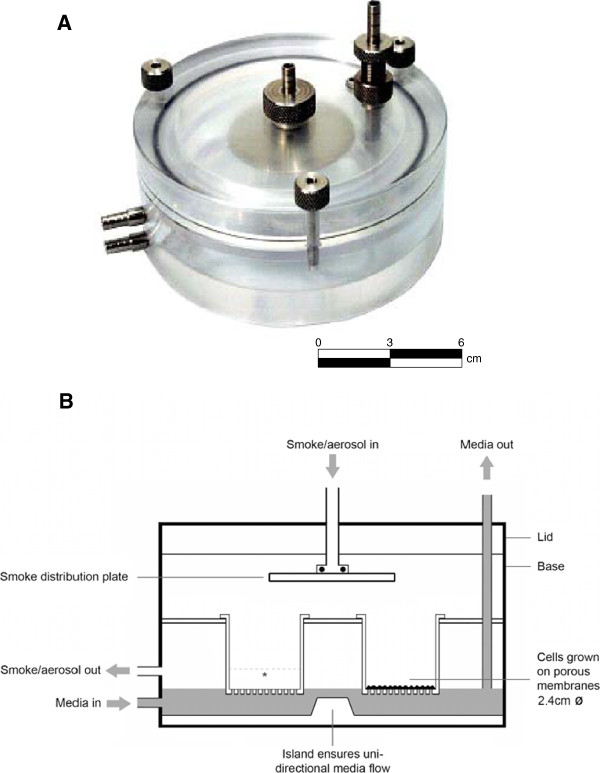
**British American Tobacco’s standard exposure chamber used for*in vitro*exposures to whole smoke at the ALI [A], and a schematic cross-section [B].** For extraction of deposited particulate matter for spectrofluorescence analysis, * illustrates the level reached by 2 ml extraction solvent when added to the exposed cell culture insert, rising 0.56 cm up the inner wall (diagram adapted from [11]).

**Figure 2:**
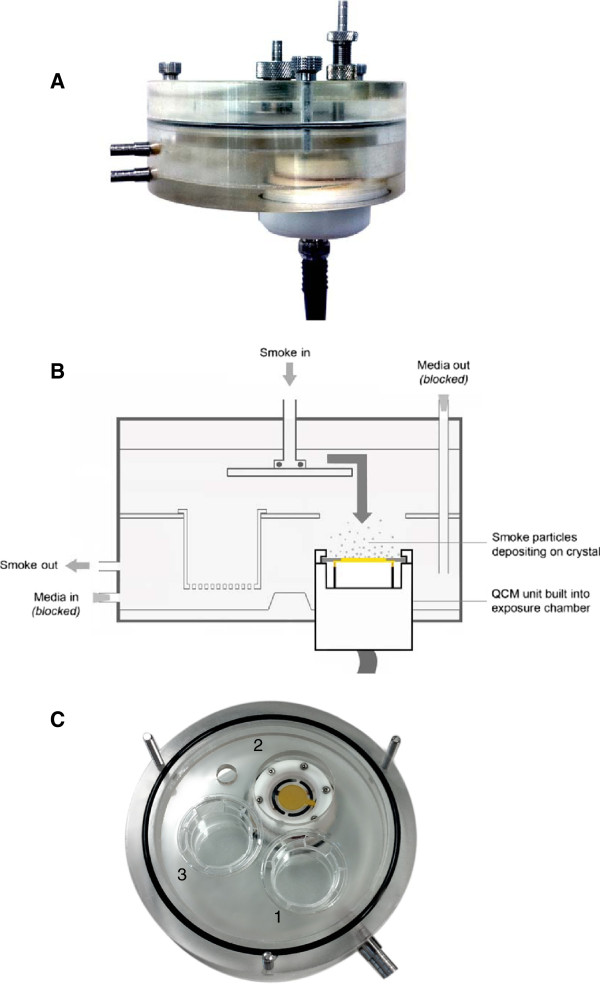
**A single QCM unit housed within the exposure chamber (side view) [A]; a schematic cross-section of the QCM exposure chamber [B]; and a top view of the chamber base showing that the QCM fits into and replaces the position of one of the three cell support inserts [C2], allowing the remaining two positions to house cell support inserts if required for parallel exposure [C1, 3].** Internal surface area and volume changes upon installation of the single QCM unit were nominal, compared to the original chamber geometry. Crystal ø = 2.5 cm; cell support insert ø = 2.4 cm; crystal’s gold electrode ø = 1.3 cm.

In this study we present a novel application of a QCM, to assess the real-time deposition of cigarette smoke *in vitro*. We have previously published studies outlining the design of an exposure chamber used to expose *in vitro* cultures at the air-liquid interface (ALI) to whole smoke (Figure 1), [2,9]. Furthermore, we have demonstrated repeatable and accurate whole smoke dilution (ranging from 1:2–1:4,000 smoke:air, volume:volume) and delivery of cigarette smoke using a commercially available Borgwaldt RM20S smoking machine (Borgwaldt-kc, Hamburg, Germany) [2,6] and successful applications *in vitro*[7,9,11]. In this study we have investigated the ability of a QCM, integrated into a whole smoke chamber (Figure 2), to detect mass differences between two different cigarette types of different tar deliveries, a 3R4F Kentucky reference cigarette (pack tar value of 9.4 mg/cigarette), and a 1 mg pack tar commercially available product. Cigarettes were smoked at various dilutions on a Borgwaldt RM20S smoking machine and real-time results on the QCM were used to quantify absolute deposition within the chamber during a 30 minute whole smoke exposure, consistent with existing *in vitro* exposure durations within our laboratories. To compare QCM real-time deposition data, a traditional chemical spectrofluorometric method to quantify particulate deposition, previously described [2], was used. 

Results from this study demonstrated the QCM was able to discriminate mass balance from two different products accurately, even on a puff-by-puff basis, within the scope of existing methodology. The integrated QCM delivered easily and reliably, real-time whole smoke mass measurements, at nanogram levels within manufacturer’s specification (at a resolution of 10 ng/cm^2^/s with a lower detection limit of 20 ng/cm^2^/h (http://www.vitrocell.com– product info download)), and demonstrated quantitative measurements and an achievable dose response. This device shows potential to be used to quantify other aerosols delivered to our chamber for *in vitro* assessment and as a possible tool for other *in vitro* exposure systems.

## Results

### QCM whole smoke quantification

**Figure 3:**
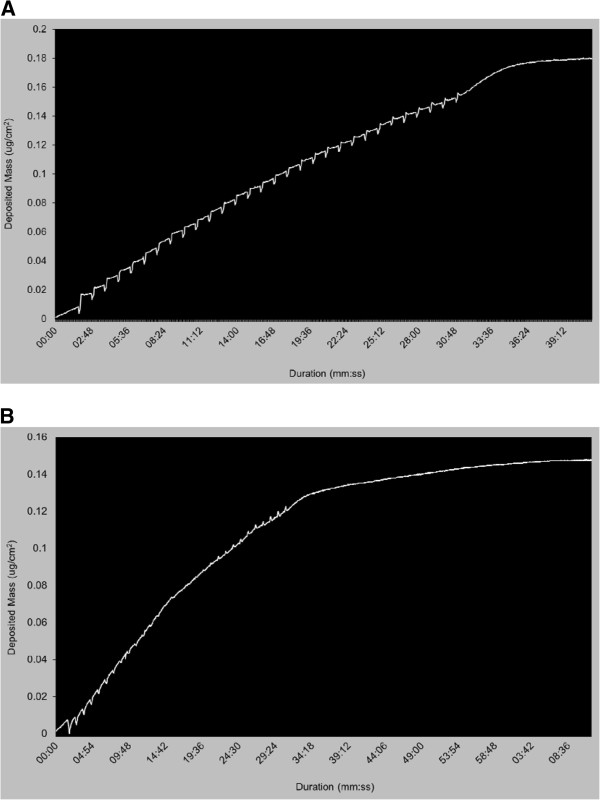
**Real-time traces of QCM deposited mass in the exposure chamber, showing a 3R4F cigarette smoked at a dilution of 1:400 [A], and a 1 mg commercially available cigarette smoked at a dilution of 1:100 [B].** Observe the plateau phase after the smoke run has finished, showing that stabilisation of the crystal is constant after approximately 10 minutes.

The QCM, integrated within the exposure chamber, was able to measure whole smoke particulate deposition on cell culture inserts within the nanogram range. Smoke was generated at a variety of dilutions from two different cigarette products. During a 30 minute whole smoke exposure, the integrated QCM could detect particulate deposition on a puff-by-puff basis with both the 3R4F reference cigarette (Figure 3A) and also the 1 mg cigarette (Figure 3B). Stable (flat) profiles were observed both before and after smoke generation. There then appeared to be a steady mass gain from first puff, followed by a repeated pattern of response per cigarette puff: a rapid initial increase in mass then a slower but more sustained increase in mass, concluding in a slight decease in mass (outlined in Figure 3A). This ‘unit’ of the repeated pattern represents one puff from the machine entering the chamber and is particularly evident with the higher tar delivery 3R4F reference cigarette, with 30 distinct repeating units per puff (Figure 3A). The mass increased with deposition of particles as smoke filled the chamber over 8 seconds, which would represent high initial deposition by turbulent mixing during filling. When the syringe smoke line valve closes after the puff has exhausted, smoke then sits in the chamber under still conditions for 52 seconds, during which a consistent slight increase in mass is observed, probably through a combination of sedimentation and diffusion. Finally, as the smoke is exhausted from the syringe and the next diluted puff enters the chamber over 8 seconds, a slight decrease in mass is observed, probably due to evaporation of some smoke vapour phase components from the surface of the crystal caused by the increase in airflow, momentarily reducing the mass. The trend is repeated per puff and the overall trend of mass increases reproducibly during exposure.

**Figure 4:**
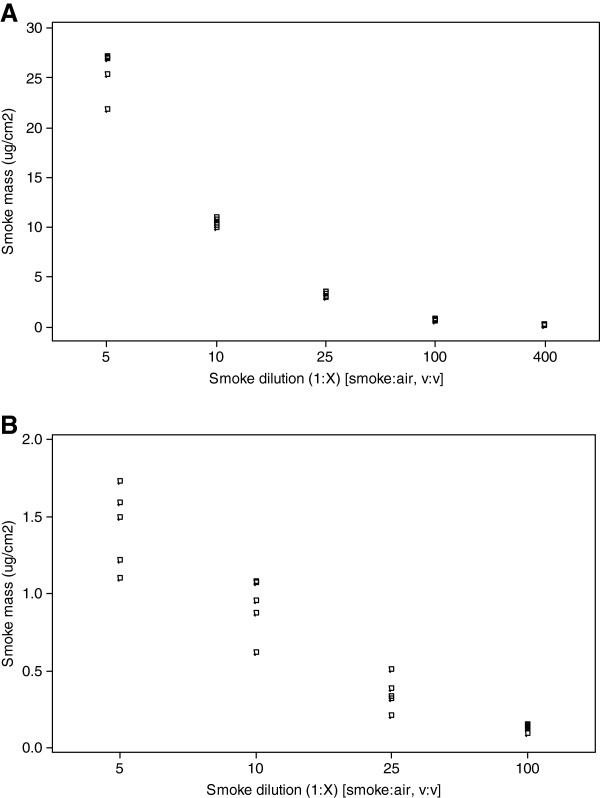
**Individual value plots showing QCM particulate deposition over a range of whole smoke dilutions tested for 3R4F reference cigarettes [A] and 1 mg commercially available cigarettes [B], for a 30 minute whole smoke exposure (n=5)**.

Both cigarette types produced an expected dose response. More particulate material was deposited at lower dilutions (higher concentrations) of smoke compared to less particulate being deposited at higher dilutions (lower concentrations) of smoke. With the 3R4F reference cigarette, 5 dilutions were studied ranging from 1:5–1:400 (smoke:air, volume:volume) (Figure 4A). Deposited mass detected within the chamber ranged from 25.75 ±2.30 μg/cm^2^ (25,750 ng/cm^2^) at the lowest dilution of smoke (1:5), to 0.22 ±0.03 μg/cm^2^ (220 ng/cm^2^) at the highest dilution of smoke (1:400) (Table 1).

**Table 1:** Particulate deposition data for 3R4F reference and 1 mg commercially available cigarettes over a range of smoke dilutions tested during a 30 minute exposure

	**mean deposited mass (μg/cm**^**2**^**)±SD**									
**Dilution (1:X)**	**5**		**10**		**25**		**100**		**400**	
**Method**	**QCM**	**Fluor**	**QCM**	**Fluor**	**QCM**	**Fluor**	**QCM**	**Fluor**	**QCM**	**Fluor**
3R4F cigarette	25.75±2.30	26.94±5.57	10.51±0.42	12.69±3.49	3.28±0.24	4.54±1.60	0.68±0.09	1.60±0.27	0.22±0.03	0.57±0.20
1 mg cigarette	1.42±0.26	1.36±0.48	0.92±0.19	0.81*≠*	0.35±0.11	0.45*≠*	0.13± 0.02	0.28*≠*	-	-

*≠ Interpolated values, taken from a standard curve of data generated within the same range but at different dilutions of 1:20, 1:50, 1:200 (smoke:air, v;v)*.

For the 1 mg commercial cigarette, 4 dilutions were studied ranging from 1:5–1:100 (Figure 4B). The highest dilution of smoke tested with the 3R4F reference cigarette (1:400) was not used for the 1 mg product as, although not lower than the absolute detection limit, it was low enough for us not to have confidence in the data obtained, based on the natural drift of the crystal, but more so due to the time taken to stabilise it prior to each run. To demonstrate, the QCM detects at a resolution of 10 ng/cm^2^/s; at the lowest dilution of 1:100 for the 1 mg cigarette the value obtained was 0.13 μg/cm^2^/60s which would notionally equate to 20 ng/cm^2^/s, close to the level of resolution. Overall, deposited mass detected within the chamber was notably less than for the 3R4F cigarette and ranged from 1.42 ±0.26 μg/cm^2^ (1,420 ng/cm^2^) at the lowest dilution of smoke (1:5), to 0.13 ±0.02 μg/cm^2^ (130 ng/cm^2^) at the highest dilution of smoke (1:100) during the 30 minutes smoke exposure (Table 1).

Standard deviation was noticeably highest at the lowest dilution of smoke (1:5) for both products tested. This may be due to the complex and concentrated nature of cigarette smoke at this dilution, the puff-by-puff measurement and cigarette by cigarette variability which is commonly observed. After every smoke run a plateau phase was observed where mass neither increased or decreased significantly, and usually took an additional 10 minutes after the smoke run. The stability at this end point demonstrated no more volatile loss and therefore robustness of the tool.

### Comparison of QCM using chemical spectrofluorometric analysis

**Figure 5:**
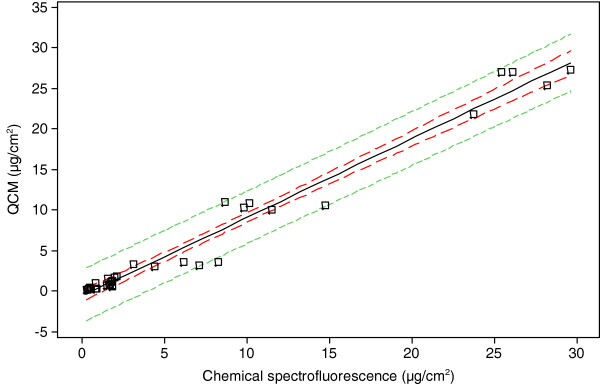
**Fitted line plot comparison of QCM and chemical spectrofluorescence assessment of whole smoke particulate deposition over a range of smoke dilutions for both 3R4F reference cigarette and a 1 mg commercially available cigarette (R**^**2**^ **= 97.4%). Solid black line = Regression, dashed red line = 95 % CI (confidence intervals), dotted green line = 95 % PI (prediction intervals).** For both cigarette types the relationship between the two methods was statistically significant (p < 0.05); this was based on a sample size large enough (n = 105) to obtain a precise estimate of the strength of the relationship. The fitted equation for the linear model which describes the relationship is: QCM = − 0.6796 + 0.9744 chemical spectrofluorescence.

To compare the utility of the QCM to routine methods, independent deposition fluorescence analyses were conducted in separate exposure chambers. Particulate matter depositing during the whole smoke exposures was eluted from the inserts and quantified using fluorescence spectroscopy. The particulate deposition data were plotted against the QCM data (in the same dilution range for each product tested) as a regression fitted line plot for both cigarette types (Figure 5).

The linear range of the results from the spectrofluorescence method was over 2 orders of magnitude for smoke dilution (1:5–1:400 for the 3R4F reference cigarette and 1:5–1:200 for the 1 mg commercial cigarette) with particle mass deliveries of 26.94- 0.57 μg/cm^2^ and 1.36- 0.28 μg/cm^2^ respectively (Table 1). As with the QCM results, the chemistry data showed that there was a positive correlation between smoke concentration and particulate depositing on the insert for both products smoked. The fitted line plot, R^2^ value was 97.4% (Figure 5) of whole smoke particulate deposition over a range of smoke dilutions for both the 3R4F reference cigarette and a 1 mg commercially available cigarette. For both cigarette types the relationship between the two methods was statistically significant (p < 0.05).”

## Discussion

We have presented a study demonstrating a novel and simple system to quantify *in vitro* cigarette smoke deposition in real-time. Although used for numerous mass measurement applications in fields such as pollution, biological, environmental and occupational monitoring [15,17,24,25], to our knowledge there is no published information on the use of the QCM for *in vitro* whole cigarette smoke assessment. A QCM was integrated into our established *in vitro* exposure chamber to quantify real-time deposition of cigarette smoke particles onto cell cultures. Furthermore, with the current switch from liquid to ALI exposures of aerosols *in vitro*[13], and increasing physiological relevance, this ALI method of particle quantification is even more important.

The QCM chamber enabled the quantification of a range of mass values per surface area for a given dilution of smoke with air, for example 25.75 ±2.30 μg/cm^2^ to 0.22 ±0.03 μg/cm^2^ mass range for 3R4F reference cigarettes dilution range 1:5–1:400 (v:v), and 1.42 ±0.26 μg/cm^2^ to 0.13 ±0.02 μg/cm^2^ mass range for 1 mg commercial cigarettes dilution range 1:5–1:100 (v:v) (Table 1). A clear dose response was demonstrated and detected by the QCM at various dilutions of cigarette smoke from two different tar delivery products (Figure 4). The QCM was sensitive enough to detect low levels of deposited matter at a resolution of 10 ng/cm^2^/s with a lower detection limit of 20 ng/cm^2^/h (http://www.vitrocell.com product info download) allowing the assessment of low 1 mg tar delivery products at high dilutions with air (Figure 3B).

The QCM data obtained in real-time were compared with a traditional chemical fluorescence method of particulate quantification; the overall distribution demonstrated a good correlation between the two described methods of particle detection, and regression analysis demonstrated that the relationship between the two methods was significant. The observed difference between the QCM and fluorescence measurements (Figure 5) could be accounted by the fluorescence method solvent extraction process, which involves multiple steps off-line and a possible effect of surface area correction to account for the total surface area of the well washed rather than just the base area of the well. It is also possible that there may be an effect of evaporation of vapour phase components from the surface of the QCM, or semi-volatiles redistributing due to a dynamic equilibrium between the particulate and vapour phase.

Deposition in the well will occur during the eight second filling period for the chamber, where turbulent mixing will dominate deposition to all surfaces and this is characterised by a sharp increase of deposited mass in the real-time QCM trace (Figure 3A). This is followed by a 52 second still period where sedimentation (to the well base) and diffusion (to all surfaces) will predominate. Calculated values [26] for a 390 nm volume median diameter smoke droplet entering the chamber at 310 K [2] represent a settling velocity of 6.28E-06 m/s with a mean displacement of 0.33 mm over 52 s. Over the same 52 s the rms displacement by diffusion is approximately 0.10 mm. These relatively small displacements are consistent with the slower rise in mass observed during the still phase. Thus we have chosen to surface area correct the spectrophotometric data for the full washed area of the well to represent the rapid deposition during mixing.

For the apparent transient mass loss at the end of the smoke residence period, two mechanisms for mass under-reporting or loss from the QCM have been described previously. The first is generally observed for the Tapered Element Oscillating Microbalance (TEOM), which shares the QCM measurement principle, albeit in a different geometry. In part, combustion particles such as diesel soot may form chain-like aggregates where only part of the chain attaches to the microbalance, hence the particle mass is not fully coupled to the crystal mass [27]. Tobacco smoke is a liquid spherical droplet [28] and as such its mass is expected to fully couple to the microbalance. As noted earlier, under ideal conditions, it is assumed that the deposited mass forms a monolayer, fully coupled to the crystal. In practice, the smoke particles are approximately 300 nm count median diameter (390 nm volume median diameter) [2], and while not initially forming a monolayer, they are sufficiently small that they would not be expected to oscillate independently of the crystal.

It has also been observed that mass loss may occur with evaporation of semi-volatile components of the aerosol mass, particularly where the balance is heated for water elimination, for example in ambient air sampling [29] and diesel sampling above. For tobacco smoke, nicotine and water, accounting for approximately 14 % of the aerosol mass for the 3R4F cigarette are effectively semi-volatile in this measurement context [30]. It has also been reported that numerous chemical species in tobacco smoke have been demonstrated to be semi-volatile in *in vitro* exposure systems [31].

Direct output of the microbalance shows puff-by-puff increases in absolute mass and a general increase in deposited mass during the inter-puff period where smoke is held in the exposure chamber. However, anomalies are observed with small transient mass losses particularly when the chamber is filling and emptying. Future work using a range of tar delivery cigarettes will help further understand the sensitivity of the QCM, but also to resolve gradient differences between products we have observed, and to discriminate deposition to the walls and base of the well.

## Materials and methods

### Whole cigarette smoke generation

Whole cigarette smoke was generated for QCM and chemical fluorescence assessment using a Borgwaldt RM20S smoking engine (Borgwaldt-kc, Hamburg, Germany) as previously described [2]. Smoke dilutions were programmed as a ratio of smoke to air; for QCM experiments 5 dilutions were chosen in the range 1:5–1:400 (smoke:air, volume:volume) (n = 5 per dilution) and for deposition experiments 7 dilutions were chosen in the range 1:5–1:400 (n = 4-6). For all experiments, the machine smoked for 30 minute duration at the ISO smoking regime (35 ml puff over 2 seconds, once a minute [32]). However, rather than smoking to standard butt length, duration was controlled by puff number; 5 cigarettes were smoked at 6 puffs each (30 minutes total). During exposure, smoke filled the chamber over 8 seconds and was then left (for 52 seconds) until the next puff of diluted smoke was delivered to the chamber - this is *batch mode* smoking, as opposed to continuous flow delivery of smoke. After machine smoking for the QCM, the device was left for an additional 10 minutes for residual smoke to settle in the chamber and the real-time deposition values to plateau. Two types of cigarettes were tested, 9.4 mg pack tar 3R4F reference cigarettes (University of Kentucky, Kentucky, USA) and 1 mg pack tar commercially available cigarettes. Pack tar refers to the value of the total particulate matter (TPM) of the cigarette smoke trapped on a Cambridge filter pad less the value of nicotine and water content; therefore pack tar is also termed ‘nicotine free dry particulate matter’ or NFDPM. Table 2 shows the characteristics of these products. Cigarettes were conditioned for a minimum of 48 hours before smoking (60 ±3 % relative humidity, 22 ±1 °C) according to ISO 3402:1999 [33]. 

**Table 2:** Physical and chemical characteristics of the cigarettes tested in this study

	**3R4F cigarette**		**1mg cigarette**	
Length (mm)	84		84	
Circumference (mm)	24.8		24.8	
Filter (mm)	27 (vented)		27 (vented)	
Ventilation (%)	29		79	
Tar^†^ (mg)	9.4*	8.8**	1.0*	0.8**
Nicotine (mg)	0.7*	0.7**	0.1*	0.1**
CO (mg)	12.0*	11.3**	2.0*	2.0**
TPM (mg)	11.0	10.4**	1.0**	
H_2_0 (mg)	0.87**		0.02**	
^†^ Nicotine- free dry particulate matter (*NFDPM)*				
* *as printed on cigarette pack*				
** *in-house analysis (n=5)*				

3R4F cigarette tar/TPM values obtained from the University of Kentucky website (http://www.ca.uky.edu/refcig).

### The QCM exposure chamber module

The previously described BAT exposure chamber [2,9] (Figure 1), was installed with a commercially available QCM unit (5 MHz AT cut quartz crystals held between two Au/Cr polished electrodes, 1 inch (2.5 cm) diameter as described by Mülhopt *et al*., 2009 [15], with associated software which converted oscillator frequency into mass per surface area (ng/cm^2^) (Vitrocell® Systems GmbH, Waldkirch, Germany), (Figure 2). The QCM read at a resolution of 10 ng/cm^2^/second, as per the manufacturer’s specification (http://www.vitrocell.com – product info download). The active area of the crystal which records deposited mass is the gold electrode in the center (Figure 2C) measuring 13 mm diameter, however the software provided converts the mass detected on this electrode to ng/cm^2^.

Before exposing to whole cigarette smoke, the QCM chamber was acclimatised to ensure quartz crystal stability for a minimum of 10 minutes at 37 °C, with the baseline re-set to zero at 1 minute increments. During the whole cigarette smoke generation and exposure phase, the QCM took mass readings every 2 seconds during the 30 minute exposure and reported as mass per unit area. Cell culture media and/or cells were not included in the chamber for these mass measurements (although capable) hence media-in and media-out ports were blocked (Figure 2B). After whole smoke exposure, quartz crystals were cleaned using 70 % ethanol and wiping with a soft lint-free tissue.

### Deposition quantification using chemical fluorescence analysis

To compare QCM generated deposition data, chemical spectrofluorescence analysis was used to quantify particulate deposition within the exposure chamber during smoke exposure at a range of dilutions generated from either 3R4F reference cigarettes or 1 mg commercially available cigarettes, as described previously [2]. These experiments were conducted independently from the QCM measurements, in separate exposure chambers and at a different time, but over the same exposure range and at exactly the same experimental conditions for whole smoke generation.

Briefly, after smoke exposure, deposited particulate material was extracted from inserts (6 well plate format) using 2 ml high performance liquid chromatography (HPLC) grade methanol (Hayman Ltd, Essex, UK) and agitation on a plate shaker at 150 rpm for 10 minutes. Extracts were analysed by HPLC using an Agilent 1100 Series (Agilent, UK). Fluorescence was detected with an Agilent standard FLD cell (Agilent, UK) at excitation and emission wavelengths of 286 nm and 350 nm respectively. Extract particulate (gravimetric particulate matter) concentrations were calculated using standard calibration curves and the blank insert results subsequently subtracted from the extract values. Data were converted to particulate deposition in mass per surface area.

Sample extracts were quantified against an external standard prepared from filter extracted particulate matter (TPM) for the 3R4F reference or 1 mg cigarettes, as described previously [2,9]. The standard calibration curve was prepared from PM concentrations ranging from 0.48 - 38 μg/ml. To compare the QCM with the spectrofluorometric data graphically, the same range of whole smoke dilutions were plotted against each other for both methods: 1:5–1:400 for the 3R4F cigarette and 1:5–1:100 for the 1 mg product.

The insert membrane diameter was 2.4 cm (6 well plate format) but with the addition of 2 ml of methanol for elution, an extra 0.56 cm (+ ≤ 0.2 cm during agitation on the plate shaker, as measured during plate agitation) of insert wall was washed (Figure 1*). This increased the potential total eluted surface area from 4.52 cm^2^ (insert membrane only) to a maximum of 10.24 cm^2^ assuming a maximum wash height of 0.76 cm. For comparative purposes, the spectrophotometric data were converted to mass per cm^2^ using the 10.24 cm^2^ area value and this was addressed further in the discussion section of this paper.

### Statistics

Data were reported as a mean ± standard deviation. Individual value plots of QCM mass (Figure 4) were created using MINITAB® v.15.1.30 statistical software, n = 5. All residual plots for all graphs were checked to ensure the quality of the data obtained. MINITAB® v.15.1.30 was also used to create the regression fitted line plots for deposition analysis using fluorescence and comparisons to QCM deposition (Figure 5); data set was at least n = 4 for all dilutions tested. Real-time traces of deposited mass (Figure 3) were made using Microsoft Excel™.

## Conclusion

This study demonstrated the QCM could successfully quantify whole cigarette smoke from two distinct products, generated and diluted using a Borgwaldt RM20S smoke engine and delivered to our whole smoke exposure chamber. The QCM quantified the mass range of delivery of whole smoke particulate from 3R4F reference cigarettes as 25.75 ±2.30 μg/cm^2^ to 0.22 ±0.03 μg/cm^2^ (within the standard dilution range of 1:5–1:400 (v:v)), and for 1 mg commercial cigarettes as 1.42 ±0.26 μg/cm^2^ to 0.13 ±0.02 μg/cm^2^ (dilution range 1:5–1:100 (v:v)). When compared to traditional chemical fluorescence method of particulate quantification the QCM measurements were statistically correlated ( (p < 0.05) with significant regression of R^2^ = 0.9744.

The QCM should be easily adapted to assess smoke mass produced from other commercially available smoking machines. Already we have applied the QCM technology to another smoking machine, the Vitrocell® VC10 Smoking Robot, with similar results. Based on the data presented here we have further developed the ‘single unit’ QCM chamber into a ‘3-in-1’ chamber with 3 identical QCM units installed, one each in the position of a cell culture insert of the BAT whole smoke exposure chamber. This expanded tool has already allowed us to assess potential positional deposition within the exposure chamber and increase replicate number per exposure. Furthermore we are currently testing the QCM module in other commercially available exposure chambers (including the Vitrocell® PT-CF mammalian exposure module) to assess its utility.

In summary, this study outlines the applicability and reliability of the QCM to assess real-time cigarette smoke particle deposition *in vitro and* suggests the QCM chamber could be a standardised measurement tool to assess and align the particle phase of whole smoke dosimetry *in vitro.* The exposure chamber alone, although designed to test cigarette whole smoke at ALI, can be used to expose *in vitro* cultures at the ALI to any aerosol, including environmental pollution, manufactured particles and fibres, aerosolised pharmaceuticals, cosmetics and pesticides or engineered nanoparticles. Therefore the scope of this chamber/QCM combination is vast.

## Abbreviations

QCM = Quartz crystal microbalance; Cmd = Count median diameter; TPM = Total particulate matter; NFDPM = Nicotine free dry particulate matter; HPLC = High performance liquid chromatography; ALI = Air-liquid interface.

## Competing interests

The authors declare that they have no competing interests.

## Authors contributions

JA conceived the study, conducted laboratory work, analysed the data and helped draft the manuscript. SH carried out the QCM work and helped draft the manuscript. DA carried out the spectrofluorometric deposition analysis and helped draft the manuscript. JMcA provided technical expertise and scientific review. MG conceived and reviewed the study and drafted the manuscript. All authors read and approved the final manuscript.
